# Mr. Whitlam, on Epilepsy

**Published:** 1805-12

**Authors:** John Whitlam

**Affiliations:** Nottingham


### *To the Editors of the Medical and Physical Journal.*

Gentlemen,

THE utility of endeavouring to find some effectual remedy for Epilepsy, must be particularly obvious to those whom practice has enabled to prove the want of efficacy in every remedy hitherto proposed for the cure of that disease.

The success attending the application of galvanism, as described in the following case, has induced me to make it public; hoping that others may experince the same advantages from the use of it.

In the month of June last, Mr. S—, a person about thirty years of age, was taken early in the morning with stupor, alternating with almost complete loss of recollection. These symptoms succeeded each other at intervals for a few minutes duration. He took a walk to a neighbouring village, thinking the fresh air might take off his indisposition. After taking a little refreshment, he was suddenly seized with an epileptic paroxysm. He fell to the ground, and continued insensible for near an hour. During his fall, he received a wound above his right eyebrow, from which it was supposed he lost half a pint of blood. When the fit left him, he felt no indisposition excepting weakness. He continued tolerably well for about a month; and then felt a return of those symptoms which had preceded his first fit. He went to the shop of a surgeon, and was bled; and as soon as the operation was ended, he rose from his seat; his voluntary powers left left him, and he again experienced a paroxysm of epilepsy.

He took some medicines; and at the end of another month, he again perceived the symptoms which had been the forerunners of his former fits. In this state he applied to me, and requested I would make use of my galvanic apparatus. The power was applied for about ten minutes; and the distressing symptoms were considerably abated. The operation was repeated in two days afterwards: and was applied the third and last time, when he felt the precursory symptoms of the disease in a more violent degree than he had ever done before. He was in a few minutes restored to the perfect use of his senses: the much dreaded fit did not return, and he has ever since been free from its effects.

The following remarks may be necessary, to elucidate the above case. The disease was never known to exist in the family of this patient. In the evening previous to his first fit, he had drank a larger quantity of ale than usual. The debility in consequence of this excess, might be an exciting cause; but perhaps, was not alone sufficient to produce the disease, as he several times since has done the same with impunity.

The machine made use of, contains 1120 square inches of surface. Two ounces of the muriatic acid were used to each pint of water used in the operation. The first and second times of its application, the power was gradually increased till very unpleasant sensations were produced. It was applied, "a capillis, usque ad unguis." The third time, from the uppermost cervical vertebræ to the feet; and then, from the same part to the extremities of the fingers.

When we consider the extensive range of the powers of Galvanism; and at the same time how much it is under the controul of the operator, we see resources in it, which are not found in pharmaceutic remedies. We can by the help of this fluid, ascend in a moment, from the mild and pleasing stimulus of the flesh-brush to the powerful one of an electric shock. We can cease to stimulate with the same facility. We can produce the effects of a rubefacient, or a blister; and where painful sensations are necessary, they may be procured instantaneously!

A description of the effects of galvanism in other diseases, will be given at another opportunity.

I am, &c.

*Nottingham, Nov. 7, 1805.* JOHN WHITLAM.

